# Marriage Reluctance Among Tunisian Women: Myth or Reality?

**DOI:** 10.1192/j.eurpsy.2025.2422

**Published:** 2025-08-26

**Authors:** D. Mezri, M. T. Ajili, A. Gharbia, U. Ouali, A. Aissa, R. Jomli

**Affiliations:** 1 Avicenne, Razi hospital, Mannouba, Tunisia

## Abstract

**Introduction:**

In recent years, Tunisia has observed a notable decrease in marriage rates, with a 36% drop in marriage contracts. This decline has been linked to post-independence reforms, modernization, the influence of tourism, media, foreign languages, rapid communication, and international migration, which have transformed values and behaviors related to marriage and family. However, it remains to be determined if the reluctance of women toward marriage is the primary cause of the decrease in marriage rates, or if other factors, such as socioeconomic conditions and male migration, are also contributing to this decline.

**Objectives:**

To determine if women are reluctant to marry.

**Methods:**

A cross-sectional study was conducted with Tunisian women using a questionnaire distributed via Google Forms and social media.

**Results:**

The study included 138 women with 121 women being not married at the time of the study. Only not married women were part of the study. The age range of the participants was from 20 to 40 years with a majority in the age range between 20 and 25 years. The majority of the participants had a university-level education (88%), came from an urban background, were active(99%); 64% were employed, 28% were university students and 7% were high school students. Interest in a serious relationship and openness to marriage was expressed by 65% of the women. 30% of women who are open to marry, reported that they are unable to find a suitable partner. Among the cases, 1.3% women were homosexual, and the lack of same-sex marriage legislation in our country was the reason they had not yet married. Additionally, 1.3% of the women admitted to having a disability or deformity, which they perceived as a challenge for a potential partner. In 16% of cases, the women expressed that economic difficulties are the reason, as they and their partners are unable to cover the costs of the wedding ceremony. However, 31% of these women reported that they are not affected by social pressure regarding marriage.

**Conclusions:**

The study indicates that reluctance to marry among Tunisian women is influenced by multiple factors rather than being a single cause. While 65% of participants expressed interest in serious relationships or marriage, challenges such as difficulty finding a suitable partner, economic constraints, lack of same-sex marriage legislation and personal disabilities or deformities contribute to the observed decrease in marriage rates. However, some women remain unaffected by social pressure. This suggests that the decline in marriage rates is a multifaceted issue, involving a combination of personal, economic, and legislative factors rather than a singular reluctance to marry.

**Disclosure of Interest:**

None Declared

